# International COVID-19 vaccine inequality amid the pandemic: Perpetuating a global crisis?

**DOI:** 10.7189/jogh.11.03086

**Published:** 2021-07-03

**Authors:** Moosa Tatar, Jalal Montazeri Shoorekchali, Mohammad Reza Faraji, Fernando A Wilson

**Affiliations:** 1 Matheson Center for Health Care Studies, University of Utah, Salt Lake City, Utah, USA; 2 Department of Population Health Sciences, University of Utah, Salt Lake City, Utah, USA; 3 Institute for Humanities and Cultural Studies, Tehran, Iran; 4 Department of Computer Science and Information Technology, Institute for Advanced Studies in Basic Sciences, Zanjan, Iran; 5 Department of Economics, University of Utah, Salt Lake City, Utah, USA

By the end of March 2021 – approximately three months after the first public COVID-19 vaccination – more than 600 million COVID-19 vaccine doses have been administered [1]. Unfortunately, there is a growing global divide in the distribution of these doses. From the beginning of the pandemic, the World Health Organization (WHO) was demanding that vaccine stockpiles be shared equitably and created the COVID-19 Vaccines Global Access (COVAX) initiative to help achieve this goal [2]. However, the wealthiest countries are being criticized for hoarding [3], in order to quickly administer the COVID-19 vaccines to their populations. In order to further understanding of this issue, we adapted Lorenz Curves and Gini Coefficients, which is a commonly utilized inequality index to illustrate the scale of the unequal distribution of COVID-19 vaccines throughout the globe [4,5].

**Figure:**
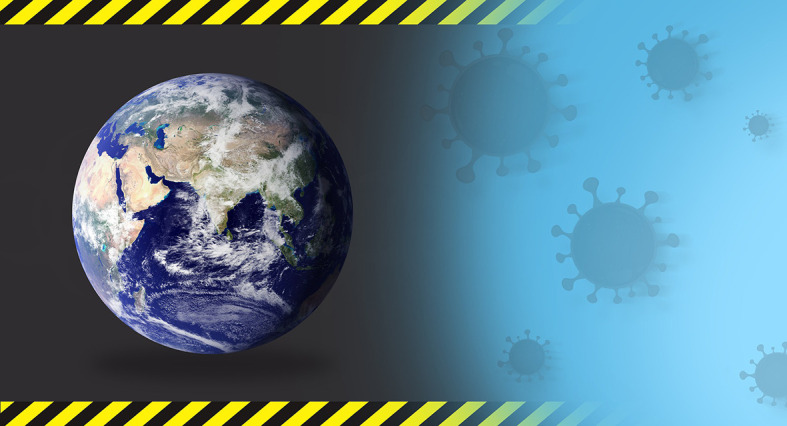
Photo: Earth Virus Attack – free image on Pixabay.

**Figure 1:**
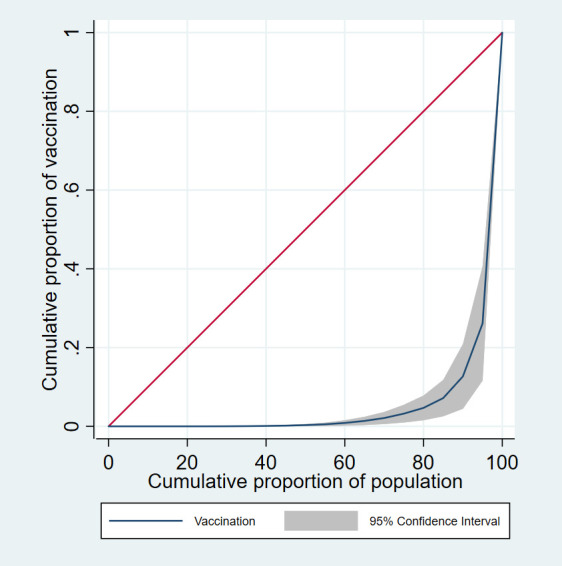
COVID-19 vaccination Lorenz curve. The cumulative proportion of vaccines is shown on the vertical (Y) axis, and the cumulative proportion of the population is shown on the horizontal (X) axis. When the vaccine distribution is perfectly equal, and the proportions of vaccines and population are equal, then equality exists, and the Lorenz curve is a straight diagonal line with a slope of 1 (45 degrees); otherwise, the Lorenz curve lies underneath the diagonal line and indicates inequality exists.

We build a data set with 178 countries, accounting for approximately 98% of the world population. Data on and COVID-19 vaccination up to March 31, 2021, were obtained from the Our World in Data website [1], and data on Gross Domestic Product (GDP) and population for 2019 (the most recent data available) were obtained from the World Bank [6,7]. **Figure 1** depicts the Lorenz Curve for COVID-19 vaccinations. The Lorenz Curve suggests a severe COVID-19 vaccine distribution inequality. In fact, 80% of the population only had approximately 5% of the total COVID-19 vaccines in the world, and the rest of the population (20%) accounted for around 95% of the COVID-19 vaccines. The Gini Coefficients for COVID-19 vaccines and GDP are 0.88 and 0.86, respectively, and express a severe COVID-19 vaccine and wealth inequality (Gini Coefficient ranges from “0” to “1”, in which “0” represents the perfect equal distribution, and “1” represents perfect unequal distribution).

Prior research suggesting extreme disparities in the COVID-19 pandemic (eg, in testing, infections, hospitalizations, and mortality) [8,9]. COVID-19 vaccination data up to March 31, 2021 indicates that the WHO's COVAX initiative has not yet been successful in the global race for COVID-19 vaccinations. In fact, several wealthy countries that signed the COVAX initiative (eg, the United States, the United Kingdom, and Germany) had a substantive share of all COVID-19 vaccines produced at the time of this study. However, total COVID-19 vaccinations may change due to reporting delays caused by the COVID-19 pandemic and may change over time, primarily because of policies that may have slowed public vaccination rollouts in some countries. The United Nations Children’s Fund (UNICEF), on behalf of the COVAX initiative, has secured two billion doses of the COVID-19 vaccine to deliver to 190 countries, including 92 low- and lower-middle-income countries and nearly 100 upper-middle-income and high-income countries by the end of 2021 [10]. This procurement and supply operation makes great strides to ensure global equitable access to COVID-19 vaccines and decrease existing global COVID-19 vaccination inequality across the world.

COVID-19 vaccine distribution is substantially unequal, and the large majority of doses have been acquired and administered in the wealthiest countries. The WHO and wealthier nations' global efforts through the COVAX initiative are vitally needed to distribute vaccines to low to middle-income countries. If not, persistent COVID-19 “hot spots” and opportunities for the emergence of new, potential “escape variants” of SARS-COV-2 may deepen the ongoing COVID-19 pandemic challenges and crisis throughout the world.
